# Exploring the perceptions of physicians, caregivers and families towards artificial nutrition and hydration for people in permanent vegetative state: How can a photo-elicitation method help?

**DOI:** 10.1371/journal.pone.0186776

**Published:** 2017-10-26

**Authors:** Elodie Cretin, Lionel Pazart, Marie-Christine Rousseau, Alain Noé, Pierre Decavel, Aline Chassagne, Aurélie Godard-Marceau, Hélène Trimaille, Florence Mathieu-Nicot, Yvan Beaussant, Damien Gabriel, Serge Daneault, Régis Aubry

**Affiliations:** 1 Clinical Investigation Center, CIC 1431 Inserm, University Hospital of Besançon, Besançon, France; 2 Palliative Care Unit, University Hospital of Besançon, Besançon, France; 3 Department of Philosophy EA 2274, University of Bourgogne Franche-Comté, Besançon, France; 4 Department of Neurosciences EA 481, University of Bourgogne Franche-Comté, Besançon, France; 5 Regional Center for Medical Ethics Bourgogne / Franche-Comté (EREBFC), Besançon, France; 6 PVS/MCS Unit, San Salvadour Hospital, AP-HP, Hyères, France; 7 PVS/MCS Unit, Center for Functional Re-education and Rehabilitation Bretignier, Héricourt, France; 8 PVS/MCS Unit, Center for Functional Re-education and Rehabilitation Les Salins de Bregille, Besançon, France; 9 Department of Socio-anthropology EA 3189, University of Bourgogne Franche-Comté, Besançon, France; 10 Department of Psychology EA 3188, University of Bourgogne Franche-Comté, Besançon, France; 11 University Hospital of Montreal, Montréal, Canada; University of Stirling, UNITED KINGDOM

## Abstract

The question of withdrawing artificial nutrition and hydration from people in a permanent vegetative state sparks considerable ethical and legal debate. Therefore, understanding the elements that influence such a decision is crucial. However, exploring perceptions of artificial nutrition and hydration is methodologically challenging for several reasons. First, because of the emotional state of the professionals and family members, who are facing an extremely distressing situation; second, because this question mirrors representations linked to a deep-rooted fear of dying of hunger and thirst; and third, because of taboos surrounding death. We sought to determine the best method to explore such complex situations in depth. This article aims to assess the relevance of the photo-elicitation interview method to analyze the perceptions and attitudes of health professionals and families of people in a permanent vegetative state regarding artificial nutrition and hydration. The photo-elicitation interview method consists in inserting one or more photographs into a research interview. An original set of 60 photos was built using Google Images and participants were asked to choose photos (10 maximum) and talk about them. The situations of 32 patients were explored in 23 dedicated centers for people in permanent vegetative state across France. In total, 138 interviews were conducted with health professionals and family members. We found that the photo-elicitation interview method 1) was well accepted by the participants and allowed them to express their emotions constructively, 2) fostered narration, reflexivity and introspection, 3) offered a sufficient "unusual angle" to allow participants to go beyond stereotypes and habits of thinking, and 4) can be replicated in other research areas. The use of visual methods currently constitutes an expanding area of research and this study stressed that this is of special interest to enhance research among populations facing end-of-life and ethical issues.

## Introduction

The progress in intensive care and resuscitation techniques in recent years has considerably increased the chances of survival after brain damage. This has led to an increasing number of patients in a vegetative state who have awakened from coma, yet remain unresponsive. In 1994, the Multi-Society Task Force on the Persistent Vegetative State [1] described this as a state of “wakefulness without awareness”, and introduced the idea of a persistent vegetative state (PVS). They also defined the temporal criteria for irreversibility (i.e., more than one year for traumatic and three months for non-traumatic (anoxic) etiology). The chances of recovery become minimal beyond these time-frames. Thus, for these persons, survival often only depends on artificial nutrition and hydration (ANH), and nursing care. Patients can survive for many years in a PVS [2]. In these situations, both the medical team and the patient’s family may have to confront ethical questions regarding the meaning of a life that is prolonged in such conditions. How far the life sustaining treatment, including ANH, should be pursued? Some consider that indefinite survival in a PVS is of no benefit to the patient and that there is no moral or legal obligation to continue life-sustaining treatments, where some consider that ANH is a primary care that is due to all human beings, especially while they are in a situation of extreme vulnerability. [3, 4].

This question has sparked fierce ethical and legal debate [5], and cases have been brought to court in several North American and European jurisdictions, such as the headline cases of Terry Schiavo in 2005 and Eluana Englaro in 2009. This debate has also been highlighted more recently in France by the case of Vincent Lambert, whose relatives are in conflict with each other regarding the question of the withdraw of artificial nutrition and hydration. On June 5^th^ 2015, the European Court of Human Rights backed France's Supreme Court decision, by agreeing that withdrawing Vincent Lambert’s gastrostomy would not violate human rights. Despite these national and European legal statements, Lambert’s parents with one of his sisters and one step brother are objecting the court decisions and are still arguing to continue the ANH, while his wife, nephew and six other brothers and sisters agree with the courts decisions. This case underscores how such ethical issues can involve factors in the decision-making process that go beyond purely medical and legal elements [4].

Indeed, the idea of withdrawing the ANH mirrors certain deeply rooted representations linked to an ancestral fear of dying of hunger and thirst, the primary act of feeding an extremely vulnerable living being, but also the taboo surrounding a medical act that will lead to death. It is therefore crucial to understand, beyond medical or legislative factors, the thought processes that influence the decision-making regarding the ethical issues of maintaining or withdrawing ANH.

However, exploring perceptions of ANH as life-sustaining care is methodologically challenging, not only for the reasons mentioned above, but also because of the emotional and psychological state of the participants [6, 7, 8]. Professional caregivers and particularly nurses taking care of those patients often suffer from burnout [9]. Similarly, the family members suffer from going through an extremely complex process of ‘impossible mourning’. Talking about the paradoxical absent presence of their loved one is extremely hard for them, and can even be impossible for some. Finally, exploring individual perceptions in this context confronts language-related problems that are very specific to the PVS situation. Indeed, these are situations where words seem to fail us: professionals and relatives often express the difficulty of describing the specific state of being of people in PVS, who are, according to their words, ‘dead and alive at the same time’ [10]. In other words, the challenge lies in putting into words situations that relate to the boundaries between life and existence [11, 12].

To explore the perceptions of physicians, caregivers and families regarding ANH in this context, we decided to perform a qualitative study using the photo-elicitation interview (PEI) method, which is known to facilitate in-depth responses when the research themes are difficult to explore [13], to give precedence to the themes that matter to the participants [14], and to be appropriate for research in vulnerable populations [15]. Our study aimed to assess the relevance of the PEI method to analyse the perceptions and attitudes of professionals and families of people in PVS regarding ANH issues. The purpose of this article is to describe and discuss the methodological adaptations that had to be made to explore the ethical issues related to PVS using a PEI method.

## Materials and methods

### What is photo-elicitation?

The photo-elicitation method is based on the simple idea of inserting a photograph into a research interview. This method was first introduced by the American anthropologist John Collier when he decided to use a new interview technique in order to address themes that researchers found difficult to explore by means of surveys or conventional in-depth interviews [13, 14, 16, 17]. Collier reported that photo interviewing involved: "*a more subtle function of graphic imagery*. *This was its compelling effect upon the informant*, *its ability to prod latent memory*, *to stimulate and release emotional statements about the informant's life*”. Moreover, he noticed that: "*the pictures elicited longer and more comprehensive interviews but at the same time helped subjects overcome the fatigue and repetition of conventional interviews*" [13].

In addition, another advantage of this method is that it improves communication during the interview: “*when two or more people discuss the meaning of the photographs they try to figure out something* together” [14]. Finally, according to Harper, the PEI makes it possible to “*break the frame*” of the participants, while also creating a bridge between the different cultural backgrounds of the researcher and the participant.

Originally used in anthropology and sociology, the PEI is increasingly used in health research [18, 19, 20, 21, 22] and among vulnerable populations [15, 23, 24, 25]. However, to the best of our knowledge, no PEI study published to date focused specifically on the issue of persons in PVS.

### Where do the photographs come from?

The PEI method can be designed and used in many different ways in the research process. An important methodological aspect lies in the question of who takes the photographs. Some projects use photos taken by the researchers themselves, while others use photos produced by the participants. The methodological decision must take into account “situated visual ethics” [26], based on the idea that the context of the photo production process itself matters as much as the contents of photos and interviews. Indeed, many contextual factors linked to this specific clinical situation must be considered before to proceeding to produce the photos.

In this study, neither the participants nor the researchers directly produced the photographs used in the PEI process. We did not ask participants to take photos for several reasons, mostly related to ethical issues. First, we were concerned about the emotional state of the people caring for the persons in PVS, especially the family members, who struggle daily with grueling incertitude, distress and social issues. Indeed, the majority of the family members who were interviewed started to cry as soon as they started to comment on the photos proposed by the researcher. In order to avoid the study being an additional cause of stress or misunderstanding, we chose not to ask the participants to take photographs of their loved ones in PVS. In addition, a photo-elicitation study complicates the traditional definition of confidentiality and consent [18, 27, 26]. If the family members were asked to take photographs, they might take pictures of people or patients not concerned by the study, which would require an additional consent process. This would be time consuming and restrictive [25], and could even lead to potential conflicts [15].

An alternative would have been to ask the healthcare professionals to take photographs, but this may also have required additional consent procedures. Furthermore, early in the preparation of the study, it was our experience that, to enhance participation of the healthcare teams, it is preferable not to ask them to perform “extra work” [25]. Lastly, apart from the obvious issues regarding anonymity in clinical care settings, asking the participants to take photographs could put them in a difficult position with an aspect that they already report to be challenging in the care of persons in PVS, namely their daily relation to the patients’ bodies. Indeed, they all reported a disturbing feeling of being intrusive and the risk of a reification of the persons in PVS, as a consequence of their extreme vulnerability: “*we cannot ask them if they agree about anything*. *This is disturbing*. *We could do anything we want to them since they are not able to protest*”. Then, asking them to take photographs of the patients could have strengthened this uncomfortable feeling.

For all these reasons, we decided not to ask the participants to take the photographs. We also decided not to use photographs taken by the researchers. During the study design phase, we were concerned that the photos might reflect only the researchers’ perspectives. We wanted to avoid both the risk of producing meaningless photos from the participants’ point of view, and photos that might be too illustrative to allow the participants to go beyond stereotypes.

We decided that a suitable solution to these challenges would be to build a set of secondary photographs using a method that has never been published to date in the available literature about PEI, namely by searching the internet for appropriate photos.

### The photo collection process using Google images

We first collected keywords related to the situation of people in PVS using a brainstorming method with health professionals who were familiar with to the care of persons in PVS but not part of the study. These keywords (Table 1) were then used to perform an advanced search of Google Images in October 2008, using the following search filters: photo content only, medium size or more, JPEG file format and moderate SafeSearch filter. Certain images were common to many key words. It was the case for the image of a tree, which appeared in Google Images results for several key words (“summer”, “winter” or “autumn”, “spring” but also for “peaceful” or “dignity” or “life”, “alive” and “end of life”). Thus, even if the word “tree” was not directly said by the participants of the brainstorming, we decided to turn the word “tree” into a key word itself and performed a secondary search for it. The same process explains the word “lake” as a key word in our list. The initial Google Images search yielded 248 photos.

**Table 1:** Key words used for the Google images search.

French key words used for the Google Images search	English translation
Absence ; Absurde ; Accident cérébral ; Accident de la vie ; Accompagner ; Alimentation artificielle; Alimentation entérale ; Amélioration ; Amour ; Appétissant ; Apport nutritionnel ; Arbre ; Arroser ; Attention ; Automne ; Autonomie ; Balance ; Bienfaisance ; Bon plat ; Cerveau ; Confort ; Culpabilité ; Décision ; Dignité ; Direction ; Discussion ; Echiquier ; Enfermée dans son propre corps ; Eté ; Ethique ; Etranger à soi-même ; Euthanasie ; Famille ; Fausse route ; Fin de vie ; Fin de vie dans la souffrance ; Fin de vie paisible ; Gastrotomie ; Gavage ; Geste médical ; Geste technique ; Gong ; Handicapé en fauteuil ; Hiver ; Hôpital ; Hydratation artificielle ; Inconfortable ; Indifférent ; Irrespectueux ; Isolement ; Lac ; Larmes ; Légume ; Libre ; Lien rompu ; Lit d’hôpital ; Lit de mort ; Mains ; Médicaments ; Mort ; Non-malfaisance ; Normale ; Nutriment ; Nutrition ; Nutrition artificielle ; Obstination déraisonnable ; Ombre ; Paisible ; Pas appétissant ; Perfusion ; Personne âgée ; Perte d’autonomie ; Pilulier ; Plante ; Pont ; Prendre la main ; Prendre soin ; Printemps ; Prise de décision ; Prison ; Projet ; Quelle direction ; Quotidien ; Regard ; Repas ; Respect de la personne ; Respect de la vie ; Sauvetage ; Semainier ; Sens de la vie ; Seule ; Sollicitude ; Sonde ; Sonde nasogastrique ; Souffrance ; Souffrir ; Stop ; Suicide ; Suicide Assisté ; Temps ; Temps qui passe ; Tourner le dos ; Traitement ; Tunnel ; Végéter ; Vers la mort ; Vie ; Vie absurde ; Vie intolérable ; Vie saine ; Vivant ; Zen.	Absence; Absurd; Stroke; life accident; Accompany; Artificial alimentation; Enteral nutrition; improvement; Love; Appetizing; Nutritional intake; Tree; Watering; attention; Autumn; Autonomy; Scales; Beneficence; good meal; Brain; Comfort; Guilt; Decision Dignity; Direction; Discussion; Chessboard; Locked into his/her own body; Summer; Ethics; Stranger to yourself; Euthanasia; Family; Swallowing the wrong way; End of life; End of life with suffering; Peaceful end of life; Gastrostomy; Forced feeding; Medical intervention; Technical intervention; Gong; Handicapped in a wheelchair; Winter; Hospital; Artificial hydration; Uncomfortable; Indifferent; Disrespectful; Isolation; Lake; Tears; Vegetable; Free; Broken relation; Hospital bed; Deathbed; Hands; Pills; Death; Non-maleficence; Normal; Nutrient; Nutrition; Artificial nutrition; Unreasonable obstinacy; Shadow; Peaceful; Not appetizing; Drip; Elderly person; Loss of autonomy; Pill box; Plant; Bridge; Holding hands; Take care of; Spring; Decision making; Prison; Project; Which direction; Daily; Look; Meal; Respect for a person; Respect for life; Rescue; Weekly calendar; Meaning of life; Alone; Solicitude; Tube; Naso-gastric tube; Suffering; Suffer; Stop; Suicide; Assisted suicide; Time; Time elapsing; Turn your back; Treatment; Tunnel; vegetate; Towards death; Life; Absurd life; Intolerable life; Healthy life; Alive ; Zen.

Among these 248 photos, the first sorting criterion was accessibility of the photos’ links and the graphic quality of the images. Photos unlinked to a web page, photos that were copyrighted or photos with poor resolution were rejected. In addition, photographs with close-up shots of faces or where specific people could be identified were either rejected or modified by blurring the faces. After this first sort, 103 photos were excluded and 145 photos remained.

A second round of sorting focused on the relevance of content, evaluated by the semi-realistic/semi—symbolic character of images. This criterion was important, in order that the photos would leave room for imagination, projections and identification. Indeed, the photographs must reflect the reality faced by the participants, while at the same time they must be sufficiently symbolic so as not to block the participant in a representation that is overly illustrative [28, 14]. Finally, a third sort of the photos was performed to achieve a balance between colour and black and white photos, and also removing any duplicates or photos sharing too many similarities.

A set of 87 photos was thus built. At this point, although 87 photos was probably too many, the number could not be reduced further through another round of sorting without running the risk of reflecting our own preferences and representations. Therefore, we decided to test this set of 87 photos with participants in a testing phase.

### Testing the photographs and comparing the PEI to other interview methods

Testing is an important and valuable step of the methodological process for studies using PEI [25]. The photograph set was tested in a 2 month testing phase with 57 participants (care-givers, physicians and relatives) concerning 9 patients in PVS, in 3 different units in France.

We were primarily concerned with the necessity of testing the photograph set with a view to reducing it, and being certain that the photos were considered appropriate from the participants’ point of view. We also needed to confirm the use of the PEI method in this study. Thus, during the testing phase, we decided to compare PEI to other conventional interview methods, to see how each method worked and to verify whether the PEI was indeed an appropriate method to explore the perceptions of the participants. Some interviews were conducted as one-to-one, semi-directed conventional interviews, while some were conducted as a one-to-one interview using PE, and finally, some focus-groups using PE were tested among health professionals only.

The focus-groups yielded interesting exchanges between professionals and a rich body of data, but did not offer the possibility to explore individual perceptions in depth, which was the goal of this study. Furthermore, this method was not retained because it was found to be difficult to organize for the research teams and extremely time-consuming. Indeed, fewer people accepted to participate in the study when focus groups were proposed. In addition, some participants said they found it difficult to express themselves freely in a group that included their co-workers or superiors.

In one-to-one semi-directed interviews, participants had obvious difficulties in putting the patient’s situation or their own emotions and perceptions into words and these interviews yielded poor content. In addition, questions about ANH and the question of whether to continue or withdraw it, made the participants (and the researcher) feel uncomfortable. Finally, this method labored under the omnipresent taboo surrounding discussions of death or the meaning of maintaining ANH.

The one-to-one PEI yielded the most in-depth individual discussions, and enhanced the quality of the relation between the researcher and the participant. Thus, the testing phase showed that the PEI was the most appropriate method for our purposes, as regards both depth of the data and the level of motivation of the participants.

### Description of the final set of photographs

In addition to validating the choice of the PEI method and to build the final set of photographs, the testing phase also aimed to test the appropriateness of the photos’ contents. Indeed, some of the contents may have been perceived as shocking or inappropriate. To this end, the participants were asked, after the interview, if they felt that any photos were inappropriate, and whether they would like to see any of the photos removed from the set.

Only one photo was designated as “shocking” by one participant (a black and white photo showing a skull in a desert) and it was thus removed from the set. Otherwise, all participants, including patients’ relatives, felt that the content of the photos was totally appropriated and bearable (sometimes despite the researcher’s own feelings that would have led to removal of certain photos). Regarding this point, the mother of a patient answered: “*compared to what we have to deal with in our loved one’s daily situation*, *nothing can really shock us anymore*”. Involving the participants and especially the relatives was important to us and contributed to reinforcing the validity of the PEI method.

Lastly, the testing phase also aimed to decrease the size of the final set of photos. During this phase, 60 of the 87 photos were all selected at least once, while 27 were never chosen at all. These 27 photos were thus removed, and the remaining 60 photos were thus retained as the final photo set, to which we added one “joker” (blank white card), in order to fill the gap in case the participants felt like a photo was “missing” [25].

A label was attributed to each photograph to guarantee a systematic and constant order of presentation of the photographs to each interviewee. The objective of labelling the photos was also to guarantee quicker identification of the photos during the interview, and to facilitate data collection by the researcher. The labels were chosen so as not to induce an idea of hierarchy between photos (e.g. “1, 2, 3, 4…”) but rather, were defined by combining letters and numbers (N1, U7, T3, R4 …) such that only the researchers could restore the original ranking order. The photos were sized to a postcard format (10x14cm), and laminated.

The final set of 60 photographs + 1 blank card contained a diversity of visual content (black and white photos, color photos, symbolic or abstract contents, realistic contents, thematic contents about food, medical environment, care-giving, family, emotions, religion, nature). This diversity is important, since one of the main advantages of the PEI method is to place the participants in favorable conditions to be able to “break their frame” [14]. The full photograph set is shown at: https://www.fc-sante.fr/cic-besancon/centre_investigation_clinique_recherche_page/nutrivege.php.

### Population

The situations of 32 persons in PVS were explored in this study, in 23 dedicated units for persons in PVS across France between July 2009 and May 2010. The 32 persons in PVS included were on average 41 years old; 20 were men, 12 were women. At the time of the study, they were considered in PVS for an average of 5 years.

A total of 138 interviews were conducted with 104 health professionals (37 nurses; 33 caregivers, 34 physicians) and 34 relatives (17 mothers; 6 husbands; 5 wives; 3 fathers; 2 daughters; 1 brother). For each person in PVS, *at least* 4 people were interviewed, namely: 1 physician, 1 nurse, 1 nurse’s aide, and 1 family member. It happened that more than 4 participants were interviewed, when people present in the unit during the study spontaneously asked to participate.

### Data collection and interview instructions

Most of the interviews took place in a quiet and isolated room, in the hospital or institution where the person in PVS was staying. At the participants’ request, 3 interviews were conducted at the patients’ bedside and one was conducted in the participant’s home (mother of a patient). The interviews lasted between 20 min and one hour. They were recorded and later transcribed in full verbatim, including non-verbal information. The participants expressed their non-opposition to participate in the study, as requested by the French law for observational clinical studies. The study protocol was approved by the Ethics Committee of the University Hospital of Besançon, France.

At the beginning of the interview, each participant was shown the set of photographs with the following instructions: "*I would like you to have a look at these photos and select the ones that evoke the situation of “Mr /Ms X” (patient’s name)*, *according to your own perception*. *There is no right or wrong answer*. *What matters is your own perception of the situation*. *You can choose as many photos as you want*, *up to 10 (from 0 to 10 photos)*. *I will then ask you to comment and explain why you chose those particular photos and what they mean to you*, *from your own point of view*."

While interviewees were commenting on the photos, the researcher wrote down the label of the selected photos and said it out loud for the recording, to be able to link the participant’s speech to the relevant photo during transcription of the interviews. After the participants had commented on all the photos they had chosen, if the question of ANH had not spontaneously been evoked by the participant through any photo they chose, semi-directed questions were then asked with the aim of exploring this specific theme.

### Analysis

Qualitative data were transcribed verbatim and organized with the support of NVIVO 9 software. Interviews were encoded by the researcher who conducted them. Data were analyzed according to qualitative research methodologies using a thematic analysis as a preliminary approach to a conceptual theorization [29, 30]. The themes thus coded were linked to the photo with which they appeared. Thus, we were able to distinguish between what was evoked spontaneously by participants while commenting the photos, and their answers to the researcher’s questions. Results were discussed within an interdisciplinary research group including: physicians, nurses, a psychologist, a philosopher and socio-anthropologists. In this article we focus on *how* themes related to ANH were, or not, evoked spontaneously by participants. The conceptual analysis of perceptions towards ANH is not presented in this article, since we focus here on the contribution of the PEI method in view of our methodological challenges.

## Findings and discussion

### Did “ANH” issues come up spontaneously when commenting on the photos?

**Fig 1:**
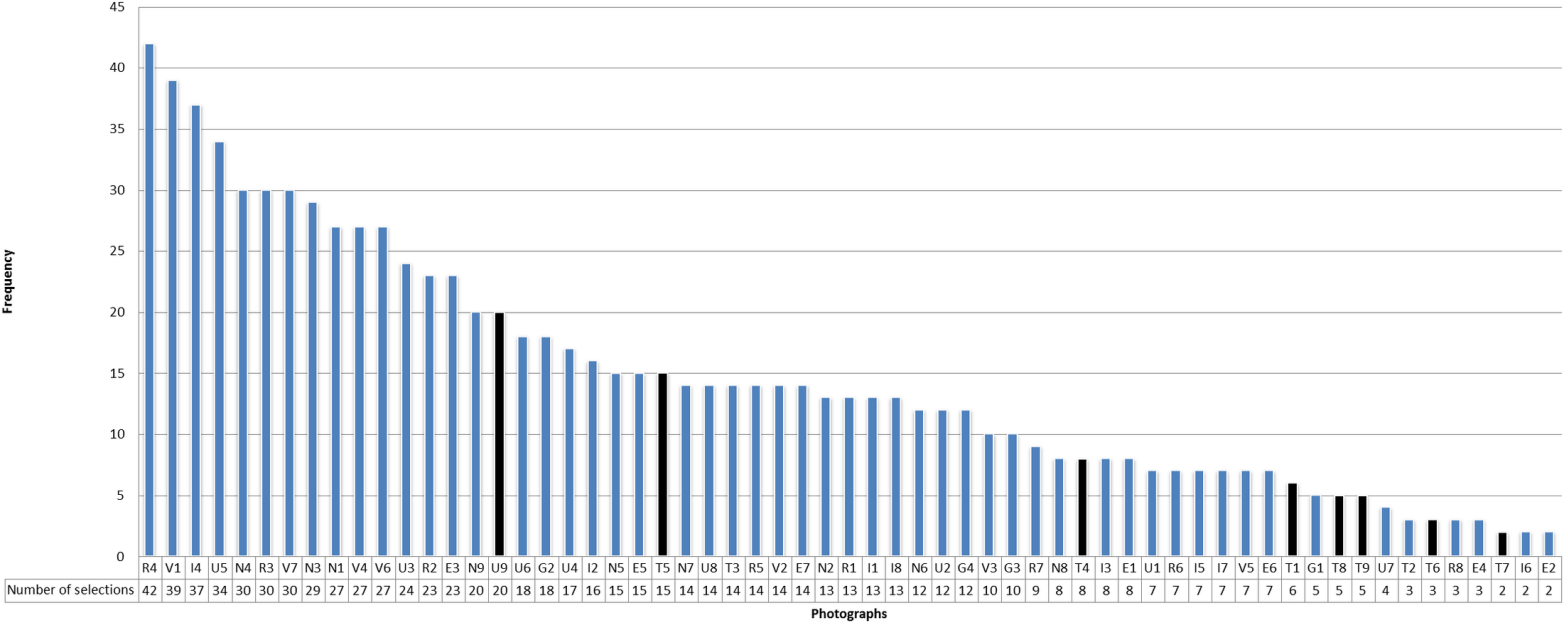
Number of selections of the 8 photos showing an explicit visual content related to food (nutrition, alimentation, hydration or drinks). The black bars indicate the 8 photos with a content related to food (photos U9, T5, T4, T1, T8, T9, T6, T7). The two most selected photos, U9 and T5 represent respectively artificial hydration or nutrition and a farmer shoving food down a goose’s throat).

**Fig 2:**
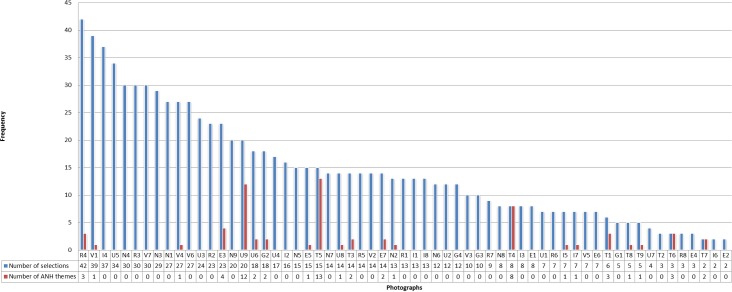
Frequency of themes related to artificial nutrition and hydration by photos. This graphic shows the photos with which the participants evoked themes related to artificial nutrition and hydration. The photo which was the most chosen to talk about artificial nutrition and hydration is the photo T5 representing a farmer shoving food down a goose’s throat.

The 8 photos whose content was explicitly related to food or ANH (included the photo of a drip, photo U9) represented 6.5% of the photos selected in the 138 interviews (selected 64 times from a total of 984 selections) (Fig 1). Those photos were also selected to talk about topics *unrelated* to artificial nutrition and hydration. Similarly, ANH themes often came up in the discussion of other photos that *did not explicitly show* food in their visual content. Finally, ANH themes were brought up 65 times through 22 photos (Fig 2).

We observed that, overall, themes related to ANH were spontaneously brought up by 34.8% of participants (Table 2). Moreover, it is noteworthy that the ANH themes were spontaneously evoked for 81.3% of the 32 patients included in the study, which means that at least one participant spontaneously talked about ANH for 81.3% of the patients included.

**Table 2:** Interviews where ANH themes where spontaneously evoked by the participants.

*Participants’ interviews*	*n*	*Interviews where "ANH" themes were evoked spontaneously by commenting a photo*	*%*
**Health professionals**	**104**	**38**	**36.5**
*Nurses*	*37*	*15*	*40*.*5*
*Nurses’aides*	*33*	*15*	*45*.*5*
*Physicians*	*34*	*8*	*23*.*5*
**Familymembers**	**34**	**10**	**29.4**
*Mothers*	*17*	*7*	*41*.*1*
*Husbands*	*6*	*1*	*16*.*6*
*Wives*	*5*	*2*	*40*
*Fathers*	*3*	*0*	*0*
*Daughters*	*2*	*0*	*0*
*Brother*	*1*	*0*	*0*
**Total**	**138**	**48**	**34.8**

The photo that was most often chosen to discuss ANH issues was photo T5, showing a farmer shoving food down a goose’s throat. We thought at first that the participants would find this photo shocking at the testing phase, but this was not the case. This may be due to the fact that this type of scene refers directly to the traditional French method of feeding geese for the production of foie gras, and is thus quite a common image for French people. Another factor in the participants’ choice of this photo is the specific word used to refer to this feeding technique. In French, feeding a goose by force is called “gavage” (force-feeding), which is also the word frequently used by nurses and physicians to refer to the practice of feeding a patient with a gastrostomy. Health professionals often said that this scene in photo T5 was disturbing, but they chose it because of the specific term “gavage”, which they use when speaking of feeding the person in PVS through their gastrostomy.

The PEI method allowed in-depth exploration of the perceptions of participants towards artificial nutrition and hydration, despite the taboo, emotional difficulties and language challenges when it came to talking about the situations of people in PVS. The participants said that this method helped them to express things that daily language fails to describe. For example:

“*There is something [in these situations] which is not… which can’t be written*, *which is intangible*” (Physician M57901s04).

“*We have to give a meaning [to these situations]*. *But it’s a meaning which has no words*. *That’s terrifying*. *It’s a meaning with no words because they [people in PVS] are life at its primitive state*. *And what is the meaning of life at its primitive state? […] We are trying to put into words a reality that cannot be put into words”* (Physician M09902s03).

### The PEI method sets the stage suitably for participants to express themselves

Compared to words-alone interviews, and in line with literature findings, the PEI method was appreciated by the participants for its original, unusual, or even "fun" aspect, [25, 31, 32] and worked as an “ice-breaker”. In a context where families and professionals often find support of PVS patients to be difficult, disconcerting and exhausting, the use of photos during the interview made the participants (and the researcher) immediately feel more comfortable, intrigued and motivated. The participants appreciated this method and frequently asked for more information about its creation and use. The medical and caregiving teams also showed an interest in using the set of photos for other purposes, outside the framework of this particular study, in order to organize discussion groups about ethical issues they have to face in their daily practice.

Moreover, by choosing and commenting on the photographs, the participants, rather than researchers, created the framework of their own interview, as they were indeed free to talk about whatever they wanted. As the photographer Diane Arbus noted [33]: “*One thing that struck me very early is that you don’t put into a photograph what’s going to come out*. *Or*, *vice versa*, *what comes out is not what you put in*”.

For the conceptual analysis, it is important not to limit the analysis to the comments where participants explain why they chose a particular photo, but one must also take into account why they did not choose other photos. Indeed, participants often mentioned the fact that they could have chosen photo T5, and why, in the end, they decided not to.

Thus, the topics spontaneously evoked with photos by the participants were those that matter to the participants, as opposed to those that matter to the researcher. Similarly, Harper suggests then that PE can be regarded “*as a postmodern dialogue based on the authority of the subject rather than the researcher*” [34, 35]. An interesting illustration of this role reversal was the use that the participants made of the “joker” card. Originally, this white blank card had been added to the photographs set in order to be used by the participants in case they feel like no photo in the set represented what they wanted to express. Chosen by 12 of the 138 participants (8 professionals and 4 family members) the joker card was actually chosen not as a joker, but because of the specific ideas the white color symbolizes itself. The participants who chose this card said that its white color was, to their eyes, evocative of a feeling of incertitude (“*It’s the void*. *We know nothing about his state*, *about his future*, *about his family’s future after his death*”—Nurse 27901s01), of the absence of the person in PVS (“*Where is she? What is she?”*—Husband 49101p01; “*This card symbolizes the emptiness*. *I see her emptied of her own self*”—Physician 18501s03) and to a feeling of peacefulness (“*I wish that I could empty my head and feel peaceful again*. *I wish that everything could be white and pure again*”–Mother 56504p01).

By letting the topics that matter to the participants come forward, the PEI makes it possible to observe the dynamics of the narrative process of each participant. These aspects were, for the goals of our study, one of the most valuable advantages of a PEI qualitative research method.

### Going beyond the language boundaries: PEI as a projective tool fostering expanding narratives

**Fig 3:**
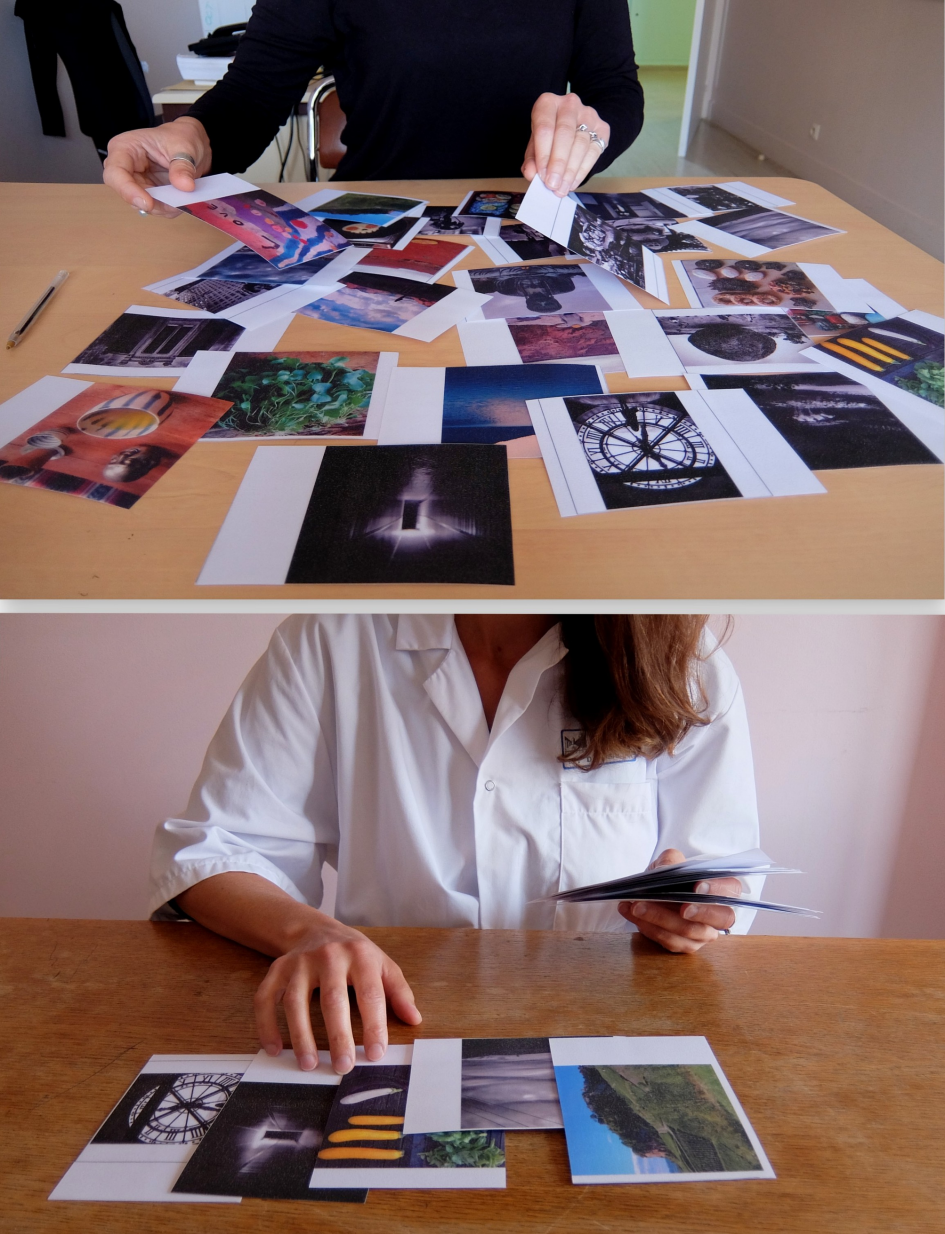
Two examples of different ways of selecting and ranking photos. This figure is similar but not identical to the one taken during the research (for illustrative purposes only).

**Fig 4:**
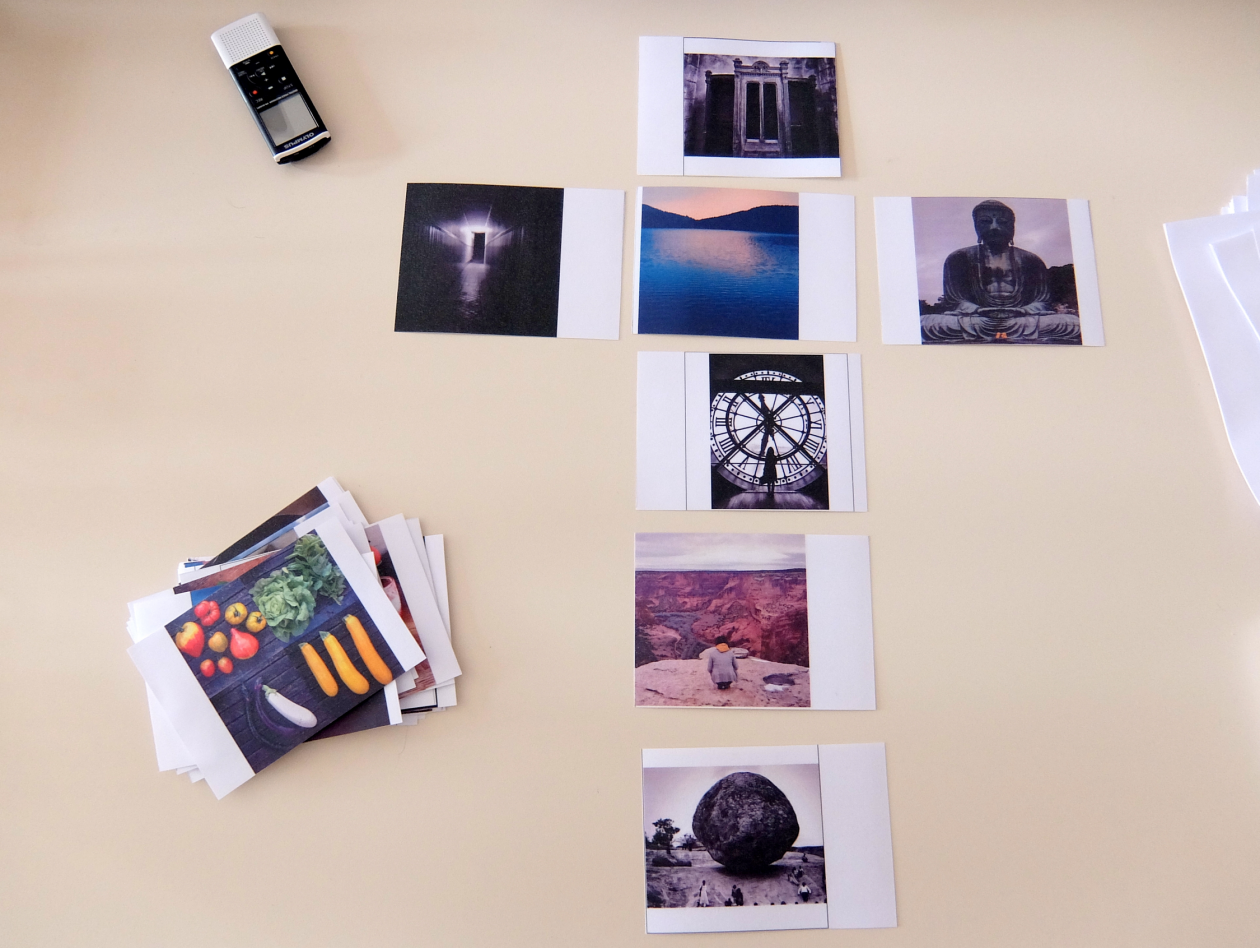
Example of a specific way to arrange the photo selection by a participant. Here, the participant explained that he purposefully arranged his photo selection in the shape of a Christian cross and built his narrative from the top to the bottom of the cross. *This figure is similar but not identical to the one taken during the research (for illustrative purposes only)*.

The PEI method offered the possibility to improve the narration, reflexivity and introspection. We expected that the time required to look over the 61 photos would be perceived as too long by the participants, but it actually turned out to be precisely enough time to allow the participants to engage directly with the subject of the study with a reflective stance. We could go so far as to say that the projective nature of the photos worked like a Rorschach ink blot, in which people can spin out their respective worlds of meaning [14]. Indeed, when talking about the contents of the photos that they had chosen themselves, the participants actually started to talk about themselves unintentionally. The narratives thus obtained were elaborated on to a lesser or greater extent, depending on the participants. However, for most interviewees, choosing and talking about the photos helped them to organize their thoughts [15] and to structure the coherence of the narration. The selection process was up to the participants: some went through a long process, made a first large selection and then picked out a few photos that they wanted to comment on while others directly selected the photos they wanted to comment on, in a quick and “efficient” manner (Fig 3). Most of the time, the participants ranked the photos they selected and built their talk like a story, with a beginning and an end. Some even accorded particular importance to the arrangement of their photo selection itself before commenting on it (Fig 4).

In addition, the PEI method presented benefits in terms of expression of emotions. Many participants (mostly relatives) were quickly overwhelmed by their emotions, and cried very early on in the interview. However, according to their own reports, the expression of those emotions was not harmful, but rather, beneficial and liberating. Indeed, they said that during the interview, they felt free to express their hopes, fears, doubts, anger, distress or satisfaction. Participants very often thanked the researcher for giving them a “platform to talk, and for listening”, which is one of the indirect benefits of any qualitative research using interviews [36]. More than just an opportunity to talk and listen, the participants expressed the sentiment that the PEI allowed them to engage in a deep reflection and introspection regarding ethical issues, which they confessed they generally preferred to avoid. A large number of participants highlighted this benefit at the end of the interviews, saying that the PEI ‘forced’ them to think, and deeply question themselves and examine their own limits, feelings and practices.

Moreover, talking about the persons in PVS via photographs was an interesting step regarding the linguistic challenge that these situations present. Indeed, finding appropriate words to describe these situations is a very specific issue. The situation of these patients eludes ordinary words, and puts people in real difficulty when it comes to describing it, and sometimes even leaves them speechless. Respondents are confronted on a daily basis with the limits of what can be said, and what can’t, and dealing with paradoxical situations where the patients are both present and absent, living but not existing. Photos made it possible for them to “capture the impossible” [14] and facilitated the use of analogies when it came to describing the patient’s state of being. Thus, the effort required to find suitable words to comment on the photos proved to be a disconcerting, but valuable exercise for both the participants’ personal reflection, and for the richness of the data collection.

### Bridging worlds by using images taken from Google images

Since the photos were not directly produced by either the researchers or the participants, but rather, came from a database containing billions of photos (indexed from the key words attributed to the photos by the persons who put them online), we can assume that, using photos from Google Images, we were able to create a common reference framework that helped to bridge the backgrounds of both researchers and participants. There was a risk that the pictures thus collected would not offer a sufficiently “unusual angle” (14) to help them to go beyond stereotypes and habits of thinking. Our results, and the participants’ feedback on the use of the PEI show that the photographs managed to bring them out of their usual framework and allowed them to improve their reflection on ethical issues concerning the situation of persons in PVS.

The use of new visual technologies or services (such as Flickr, Facebook, Instagram, YouTube, Twitter, blogs) currently constitutes an expanding area of research [37, 38]. In this context, our experience shows that using image databases like Google Images is an efficient tool, as long as the methodological process takes account of the specific ethical and situational factors related to the project’s context and goals.

Our photo collection process can easily be reproduced by other research teams using a brainstorming method coupled with searches of Google Images. Thus, this method could be further explored in a variety of contexts. It could also offer the possibility of cross-cultural studies, based on a collectively constructed set of photographs.

It would also be of interest to develop an electronic version of a set of photographs in the framework of studies including a large population. In particular, a website could be made available to visualize the photos, make a selection and record written comments from participants. However, such “online” use of the PEI would not allow in-depth responses, since the face-to-face relationship between the research and the participant would be lost.

## Conclusion

Our results suggest that the PEI method is appropriate to explore and record representations of the persons involved in the care of people in PVS. Indeed, this approach fosters investment, reflection and expression of respondents in a context of multiple, challenging obstacles, such as a difficult emotional context and language-related issues. Using Google Images proved to be an efficient method to collect images that made it possible to bridge the backgrounds of both researchers and participants, while offering sufficient diversity to “break the frame” of participants and yield in-depth interviews. Our experience showed that using image databases like Google Images is an efficient tool, as long as the methodological decision process takes account of the specific ethical and situational factors related to the research goals.
